# Association between maternal acculturation and health beliefs related to oral health of Latino children

**DOI:** 10.1186/s12903-018-0532-4

**Published:** 2018-04-24

**Authors:** Tamanna Tiwari, Matthew Mulvahill, Anne Wilson, Nayanjot Rai, Judith Albino

**Affiliations:** 1 Department of Community Dentistry and Population Health, School of Dental Medicine, University of Colorado Anschutz Medical Campus, Aurora, Colorado USA; 2 School of Medicine, University of Colorado Anschutz Medical Campus, Aurora, Colorado USA; 3 Colorado School of Public Health, University of Colorado Anschutz Medical Campus, Aurora, Colorado USA; 4 Center for Native Oral Health Research, University of Colorado Anschutz Medical Campus, Aurora, Colorado USA

**Keywords:** Oral health knowledge, Acculturation, Health belief model

## Abstract

**Background:**

This report is presenting the association of maternal acculturation, measured by preferred language, and oral health-related psychosocial measures in an urban Latino population.

**Methods:**

A cross-sectional survey was conducted with 100 mother-child dyads from the Dental Center at the Children’s Hospital Colorado, the University of Colorado. A portion of Basic Research Factors Questionnaire capturing information about parental dental knowledge, attitudes, behavior and psychosocial measures was used to collect data from the participating mothers. Descriptive statistics were calculated for demographics and psychosocial measures by acculturation. A univariate linear regression model was performed for each measure by preferred language for primary analysis followed by adjusted model adjusting for parent’s education.

**Results:**

The mean age of the children was 3.99 years (SD = 1.11), and that of the mother was 29.54 years (SD = 9.62). Dental caries, measured as dmfs, was significantly higher in children of Spanish-speaking mothers compared to children of English-speaking mothers. English-speaking mothers had higher mean scores of oral health knowledge, oral health behaviors, knowledge on dental utilization, self-efficacy, and Oral Health Locus of Control as compared to Spanish-speaking mothers. Univariate analysis demonstrated significant association for preference for Spanish language with knowledge on dental utilization, maternal self-efficacy, perceived susceptibility and perceived barriers. The effect of language was attenuated, but significant, for each of these variables after adjusting for parent’s education.

**Conclusion:**

This study reported that higher acculturation measured by a preference for the English language had a positive association with oral health outcomes in children. Spanish-speaking mothers perceived that their children were less susceptible to caries. Additionally, they perceived barriers in visiting the dentist for preventive visits.

## Background

Acculturation is defined as the process by which individuals from a community may adopt the values and behaviors from another culture, and this may, in turn, affect their beliefs [1]. According to recent literature, acculturation is considered a multidimensional process, in which sociocultural changes may occur throughout the person’s life course and varying even for individuals living within the same community [1–3].

There are many potential frameworks to understand the acculturation process and how it impacts health in immigrant populations. According to one theoretical framework, migration may cause psychosocial distress or loss of social support, resulting in a new behavioral environment that could be associated with the level of acculturation for an individual [3]. The framework presented by Fox et al. describes how socioeconomic conditions of migrant families may influence higher assimilation into the host culture, thereby resulting in higher levels of acculturation [3]. Furthermore, length of stay, language spoken at home, and social networks may influence the level of acculturation for an individual or a family [4, 5]. For example, educational level, preferred language, and level of dental knowledge of the social networks are associated with a higher level of acculturation and, in turn, with the utilization of dental health services [6].

Another concept presented by Gao et al. discusses that the impact of acculturation on health can be mediated via a change in health behaviors and perceptions of one’s ability to prevent disease or receive treatment [7]. In the context of health beliefs, acculturation can influence utilization of health services, doctor-patient communication, adopting new health behaviors, and treatment decisions [7, 8]. Furthermore, acculturation may have a positive or negative effect on the health outcome in question [1]. For example, acculturation can be a risk factor for adoption of behaviors such as increased alcohol consumption, smoking, and higher fast food consumption, and on the other hand, it may promote increased amount of time engaged in exercise and physical activity [2].

For oral health outcomes, acculturation has been seen to influence the oral health behaviors, access, and navigation of oral healthcare [9, 10]. A recent systematic review of the impact of acculturation on oral health concluded that though higher levels of acculturation had a positive effect on utilization of dental services, this did not necessarily lead to improving oral health outcomes [7]. The authors also concluded that lower levels of acculturation might increase the risk of uptake of some unhealthy oral health-related behaviors, such as consumption of high sugary foods and drinks, and increase the risk of poor oral health outcomes [7]. Although there is a growing recognition that acculturation is a complicated process and that it influences oral health by a complex interplay of psychosocial factors, there is a scarcity of dental research addressing these issues.

Latinos are the largest and fastest immigrant group in the US, with a growth of 39% in Latino children between 2000 to 2010 [8]. In addition, within the 21% US households that speak a language other than English, 62% of these households are Spanish speaking [11]. Thus this study aimed to examine the association of maternal acculturation, measured as preferred language, and oral health-related psychosocial measures in an urban Latino population.

## Methods

A cross-sectional survey was conducted with 100 mother-child dyads. One hundred dyads consisting of Latina mothers who were at least 18 years of age with a child under the age of 6 years were enrolled at the Dental Center at Children’s Hospital Colorado, the University of Colorado in Aurora, Colorado. Latino families make up about 50% of the patient population at the Dental Center. The study only enrolled mothers who are primary caregivers to maintain reliability in the same kind of caregivers they study. A convenience sample of Latina mothers was enrolled from the waiting room of the Dental Center at Children’s Hospital. A co-investigator on the study prescreened the mothers, and because Latina mothers were eligible for the study, pre-screening enabled appropriate enrollment. Information about the study was provided in English and Spanish, with a consent form detailing the approach of the study. Participating mothers were given the option to hear about the study details, sign the consent form and complete the survey in English or Spanish. Certified translators provided study information to Spanish-speaking mothers. This study was approved by the Colorado Multiple Institutional Review Board (COMIRB).

### Measures

A manuscript with the main outcomes and study methodology has been recently published and presents all the details of the study design and implementation [12]. The questionnaire used in this study is a portion of the Basic Research Factors Questionnaire (BRFQ) [13] (Table 1), which captures parental dental knowledge, attitudes, behaviors, and other psychosocial measures. The BRFQ was developed as a collaborative effort involving three Centers for Research to Reduce Oral Health Disparities: located at the University of Colorado Denver, Boston University, and the University of California San Francisco (UCSF), and funded by the National Institute of Dental and Craniofacial Research. The BRFQ is available in English and Spanish and is being used by all three centers in diverse populations.

**Table 1:** Description of Measures

Measure	Description
Oral Health Locus of Control (OHLOC) 3 subscales	OHLOC captures a person’s attitudes about who or what has control over their child’s oral health outcomes. There are 3 subscales, which represents the extent to which participants believe control of their child’s oral health outcomes lies with the parent (internal LOC), the dentist (powerful other LOC), or chance factors (chance LOC).
Health Belief Model 4 subscales	The Health Belief Model is one of the major models that have been used to explain health behavior. The model predicts that behavior is a function of 4 subscales - Perceived susceptibility, Perceived severity, Perceived barriers, Perceived benefits.
Self-Efficacy	Self-efficacy represents a person’s confidence that he/she can successfully engage in a specific health behavior. Self-efficacy score represents how sure participants are that they can engage in recommended behavior to take care of their children’s teeth. Ten questions quantify this measure.
Oral Health Behavior	The overall behavior score represents the percentage of 13 oral health behavior items that were answered with an adherent response, where “adherent” means the participant engages in the recommended oral health behavior.
Oral Health Knowledge	The overall knowledge score represents the percentage of (how many) oral health knowledge items answered correctly. Nineteen questions quantify this measure.
Knowledge on dental utilization	Five items measured parental knowledge on utilization of oral health services for their children.

All the children participating in the study received an oral examination by one of the co-investigators of the study. Decayed, missing, filled, surfaces (dmfs) were measured during this examination. Data was recorded using an electronic dental research recording instrument designated as CARIN (Caries Research Instrument), specifically designed for research documentation involving dmfs.

Table 1 provides the description of the measures and the subscales of the BRFQ used in this study. Psychosocial measures include oral health behavior, oral health knowledge, knowledge on dental utilization, self-efficacy, three subscales on Oral Health Locus of Control, and four subscales from the Health Belief Model (Table 1). Knowledge on dental care utilization is another variable that measures the mother’s knowledge about when to take the child for dental care, preventive visits vs. visiting the dentist in response to pain and the impact of her friends and family on her dental care utilization for her child. Behavior and oral health knowledge scales range from 0 to 100 and were calculated as the percent of individual items marked correctly. The remaining measures utilized Likert-type scales, with scores on each item ranging from 1 to 5. The summary measures are the average of the values for answered questions, thus also ranging from 1 to 5.

Language preference was used as a proxy for acculturation in this study. Several studies have used this proxy measure to capture acculturation, as use of non-native language has been associated with higher utilization of health services and better health in immigrant populations [5, 14]. Preference for English or Spanish was measured by asking the participating mothers if they wanted to complete the consent form and study survey in English or Spanish. Spanish-speaking mothers were considered to be less-acculturated or closer to the Latino culture, as compared to English- speaking.

### Data analysis

Analyses for this study tested the effect of preferred language, which was considered a proxy for acculturation, on psychosocial measures of oral health. Descriptive statistics were calculated for demographics and psychosocial measures by acculturation. Descriptive statistics presented for continuous variables are both mean (SD) and median (IQR), where IQR is the 25% and 75% or inter-quartile interval. For categorical variables, count and percentages are presented. The primary analysis consists of univariate linear regression models for each measure by preferred language, as well as a model adjusting for parent’s education. A significance level of 0.05 was used in all hypothesis testing and confidence intervals. Data cleaning and analysis were conducted using R version 3.3.2 (2016–10-31) (R Core Team 2013).

## Results

Table 2 provides demographic characteristics of the study sample, presently separately by the preferred language of communication. Fifty-nine mothers were English speaking, and 40 were a Spanish speaker. The mean age of the children in the study was 3.99 years (SD = 1.11), and that of the mother was 29.54 years (SD = 9.62). Dental caries, measured as dmfs, was significantly higher (15.20 ± 21.48) in children of Spanish-speaking mothers than for children of English-speaking mothers (7.56 ± 12.11) (*p* = 0.043).

**Table 2:** Descriptive statistics for demographic variables by acculturation

	language: English (*N* = 59) mean (SD)	language: Spanish (*N* = 40) mean (SD)
Gender		
Male	32 (54.24%)	21 (52.50%)
Female	27 (45.76%)	19 (47.50%)
Child’s Age	3.94 ± 1.09	4.05 ± 1.15
Mother’s Age	28.20 ± 9.77	31.84 ± 9.05
Mother’s Education		
Less than HS	17/56 (30.36%)	20/38 (52.63%)
HS or more	39/56 (69.64%)	18/38 (47.37%)
Mother’s Employment		
Employed	23 (38.98%)	12 (30.00%)
Not employed	36 (61.02%)	28 (70.00%)
Household Size	4.66 ± 1.53	4.89 ± 1.23
Household Income		
$10,830 - $14,569	3/28 (10.71%)	1/13 (7.69%)
$14,570 - $18,309	3/28 (10.71%)	1/13 (7.69%)
$18,310 - $22,049	2/28 (7.14%)	3/13 (23.08%)
$22,050 - $25,789	3/28 (10.71%)	2/13 (15.38%)
$25,790 - $29,529	12/28 (42.86%)	3/13 (23.08%)
$29,530 - $33,269	0/28 (0.00%)	2/13 (15.38%)
$33,270 - $37,009	3/28 (10.71%)	1/13 (7.69%)
$37,010	2/28 (7.14%)	0/13 (0.00%)
Household Minors	2.61 ± 1.42	2.72 ± 1.28
Household Years in Household	4.58 ± 3.67	3.79 ± 3.25
Dental Caries (dmfs)	7.56 ± 12.11	15.20 ± 21.48

Table 3 provides the means of the psychosocial measures by preferred language. English-speaking mothers had higher mean scores of oral health knowledge (87.51 ± 7.65), behaviors (47.13 ± 14.98), knowledge of dental utilization (3.67 ± 0.51), and self-efficacy (4.34 ± 0.59), compared to Spanish-speaking mothers. English-speaking mothers had higher scores of Internal Oral Health Locus of Control (4.38 ± 0.68); Spanish-speaking mothers scored higher on one subscale of the Health Belief Model, Perceived Barriers (4.07 ± 1.25).

**Table 3:** Descriptive statistics for psychosocial measures by acculturation

	language: English (*N* = 59)	language: Spanish (*N* = 40)
Oral health Behavior (*P* = 0.0988)		
mean (SD)	47.13 ± 14.98	41.76 ± 16.18
median (IQR)	50.00 (34.85, 58.33)	41.67 (31.82, 50.00)
Oral health knowledge (*P* = 0.3995)		
mean (SD)	87.51 ± 7.65	85.67 ± 12.18
median (IQR)	88.89 (82.35, 93.54)	88.24 (82.35, 94.12)
Knowledge on dental utilization (*P* = 0.0024)		
mean (SD)	3.67 ± 0.51	3.15 ± 0.93
median (IQR)	3.60 (3.40, 4.00)	3.40 (2.45, 4.00)
Self-efficacy (*P* = 0.0043)		
mean (SD)	4.34 ± 0.59	3.91 ± 0.79
median (IQR)	4.50 (4.00, 4.80)	4.06 (3.60, 4.45)
Locus of control - Internal (*P* = 0.1655)		
mean (SD)	4.38 ± 0.68	4.07 ± 1.25
median (IQR)	4.67 (4.00, 5.00)	4.67 (3.67, 5.00)
Locus of control - External Others (*P* = 0.6971)		
mean (SD)	2.19 ± 1.01	2.27 ± 0.94
median (IQR)	2.00 (1.33, 2.67)	2.33 (1.67, 3.00)
Locus of control - External Chance (*P* = 0.6227)		
mean (SD)	2.10 ± 1.01	2.22 ± 1.19
median (IQR)	2.00 (1.33, 3.00)	2.00 (1.00, 3.00)
Health belief model - Severity (*P* = 0.0574)		
mean (SD)	4.29 ± 0.94	3.94 ± 0.82
median (IQR)	4.67 (3.67, 5.00)	3.67 (3.58, 5.00)
Health belief model - Barriers (*P* = 0.0002)		
mean (SD)	1.89 ± 0.61	2.42 ± 0.68
median (IQR)	1.80 (1.45, 2.20)	2.45 (1.80, 3.00)
Health belief model - Susceptibility (*P* = 0.0080)		
mean (SD)	3.34 ± 0.75	2.83 ± 1.03
median (IQR)	3.50 (3.00, 3.75)	3.00 (2.00, 3.50)
Health belief model - Benefits (*P* = 0.1951)		
mean (SD)	4.31 ± 0.67	4.03 ± 1.24
median (IQR)	4.40 (4.20, 4.80)	4.55 (3.70, 5.00)

In the univariate models (Table 4), the preference for Spanish-language was significantly associated with knowledge of dental utilization (− 0.51, *P* = 0.0006), maternal self-efficacy (− 0.44, *P* = 0.0024). Preference for the Spanish language was also significantly associated with maternal Perceived Susceptibility (− 0.52, *P* = 0.0046) Perceived Barriers (0.53, *P* = 0.0001), and two subscales of Health Belief Model. After adjusting for parent’s education, the effect of language was slightly attenuated, though still significant, for each of these variables, suggesting that parent’s education was a significant confounder.

**Table 4:** Linear regression of each psychosocial variable on primary language (Spanish) on both a univariate basis and adjusted for parent’s education

Outcome	estimate^*^	*p*.value	Edu Adj. estimate^*^	Edu Adj. *p*.value
Oral health Behavior	−5.37 (−11.66, 0.92)	*P* = 0.0932	−5.69 (−12.21, 0.84)	*P* = 0.0867
Oral health Knowledge	−1.84 (− 5.79, 2.11)	*P* = 0.3581	− 1.41 (− 5.45, 2.63)	*P* = 0.4894
Knowledge on dental service utilization	−0.51 (−0.80, −0.22)	*P* = 0.0006	−0.41 (− 0.71, − 0.11)	*P* = 0.0073
Self-efficacy	− 0.44 (− 0.71, − 0.16)	*P* = 0.0024	− 0.35 (− 0.62, − 0.09)	*P* = 0.0099
HBM - Severity	−0.34 (− 0.71, 0.02)	*P* = 0.0636	−0.26 (− 0.65, 0.12)	*P* = 0.1800
HBM - Barriers	0.53 (0.27, 0.79)	*P* = 0.0001	0.46 (0.20, 0.71)	*P* = 0.0006
HBM - Susceptibility	−0.52 (− 0.87, − 0.16)	*P* = 0.0046	−0.41 (− 0.78, − 0.05)	*P* = 0.0273
HBM - Benefits	−0.28 (− 0.66, 0.10)	*P* = 0.1477	−0.20 (− 0.57, 0.18)	*P* = 0.3045
LOC - Internal	−0.30 (− 0.69, 0.08)	*P* = 0.1213	−0.20 (− 0.57, 0.17)	*P* = 0.2867
LOC - External Others	0.08 (− 0.32, 0.48)	*P* = 0.7012	0.07 (− 0.36, 0.49)	*P* = 0.7600
LOC - External Chance	0.11 (− 0.33, 0.56)	*P* = 0.6114	0.10 (− 0.38, 0.58)	*P* = 0.6715

*Estimates are the difference in the psychosocial measure from primarily English-speaking and Spanish-speaking patients

## Discussion

The impetus for acculturation research in health is to determine whether experiences of a particular group have effects on trends in health outcome [15]. In this study, higher acculturation, which was measured by a preference for the English language had a positive association with oral health outcomes, i.e., dental caries in children. Children of English-speaking mothers had significantly lower dmfs scores compared with children of Spanish-speaking mothers. Spanish-speaking mothers had low knowledge of dental care utilization, low self-efficacy; they perceived their children not susceptible to dental caries and perceived more barriers in visiting the dentist for prevention visits.

Our assumption was that recruiting a health center population should have neutralized any potential structural barriers related to utilization or access to care. However, Spanish-speaking mothers in this study perceived barriers in visiting the dentist for preventive visits and had poor oral health behaviors towards their children, when compared with English-speaking mothers. They also had perceptions that their children are less susceptible to developing dental caries, an attitude that may suggest why they did not do much about preventing dental caries. Spanish-speaking mothers had less knowledge related to dental care utilization, including timing and necessity of a dental visit, and they submitted to the notion that young children should only be taken for a dental visit if they were in pain. Furthermore, their social networks, including family and friends, supported not visiting the dentist unless there was an acute need, and they agreed that no one in their family told them the child should be taken for a preventive dental visit.

These perceptions could be related to cultural norms and beliefs, and because Spanish-speaking mothers were less acculturated compared with English-speaking mothers, their beliefs related to preventive dental visits would be inclined towards the Latino cultural norms [8], which in one study that was conducted with Mexican Americans has been described as a “reactive orientation” towards dental care [4]. Previous research also has shown that Latino parents can have fatalistic attitudes towards the oral health of their children and reduced utilization due to perceived barriers [16, 17]. Another study that collected qualitative data from Spanish-speaking Latina mothers at the same health center reported that they struggled to overcome the familial pressure against visiting the dentist for preventive appointments for their children and received suggestions that dental visits should be done in response to pain [18].

These differences in maternal oral health beliefs and behaviors can be embedded theoretically in the level of assimilation into the Anglo culture [2]. For example, partial acculturation of Latino families-- that is, alienation from their traditional culture and incomplete integration into the new culture, may put them at greater risk for poor oral health related behaviors, which may also impact utilization and ultimately disease outcome [7, 8].

Although acculturation was measured by the proxy variable of preferred language use, it still shows an influence on maternal beliefs and perceptions related to caries prevention and dental care utilization -- and ultimately the dental caries experience in children in this study. Lacking the ability to speak English can be seen as an external manifestation of “slow cultural transformation,” which may influence access to care and navigation of the healthcare system, thus indirectly affecting health outcomes [19]. Within pediatric oral health research, lower dental care utilization, lower sealant applications and delayed first dental visits have been related to non-English speaking households [8]. For non-English speaking mothers, it may become difficult to follow the oral hygiene instructions and recommended behaviors given at the dental office, and because of the language barrier, their social networks might be limited to Spanish-speaking individuals. Recent studies have shown the complex role of social networks and acculturation in oral health [4]. Moreover, it has been reported that reduced English skills may cause significant difficulties for Latino families, making them less trusting towards dental care professionals and not establishing a dental home [10, 20].

The present study has some limitations. We drew our sample from a conveniently available urban health center population that may not be generalizable to the larger population. Acculturation was measured using the proxy variable of language preference, and we did not collect information on the birthplace of the mother if she was U.S. born or foreign born.

## Conclusion

This study makes important contributions to understanding the underlying psychosocial and behavioral factors that predict how acculturation impacts oral health. Further longitudinal research is warranted in this direction, in using a multi-dimensional tool to measure acculturation rather than using a proxy variable. The author has secured funding to conduct a study that will use a multidimensional scale to measure acculturation.

## Abbreviations

BRFQ: Basic Research Factors Questionnaire
IQR: Interquartile Range
SD: Standard Deviation
UCSF: University of California San Francisco
